# Modeling Alcohol’s Effects on Organs in Animal Models

**Published:** 2000

**Authors:** Biddanda C. Ponnappa, Emanuel Rubin

**Affiliations:** Biddanda C. Ponnappa, Ph.D., is an associate professor and Emanuel Rubin, M.D., is a professor and chairman in the Department of Pathology, Anatomy and Cell Biology, Thomas Jefferson University, Philadelphia, Pennsylvania

**Keywords:** animal model, chronic AODE (effects of AOD [alcohol or other drug] use, abuse, and dependence), in vitro study, body part, body fluid, alcoholic liver disorder, alcoholic cardiomyopathy, fetal alcohol syndrome, alcohol-related neurodevelopment disorder, ethanol metabolism

## Abstract

Researchers have developed numerous animal models to investigate the development of various alcohol-related diseases. Such models have provided insights into the mechanism through which alcohol can induce liver damage. Animal models also have helped researchers explore the mechanisms by which both short-term (e.g., binge) and long-term drinking can interfere with the function of the heart, a condition referred to as alcoholic cardiomyopathy. Furthermore, animal models have provided substantial information on the causes of fetal alcohol syndrome. Such models have demonstrated that exposure to alcohol during gestation can lead to prenatal and postnatal growth retardation, characteristic facial malformations, immune system deficiencies, and alterations in the central nervous system.

Long-term (i.e., chronic) alcohol use affects almost every organ system of the body, potentially resulting in serious illnesses, including liver disease, impaired heart function (i.e., cardiomyopathy^1^), and inflammation of the pancreas (i.e., pancreatitis). Even one-time (i.e., acute) alcohol consumption, such as binge drinking, can temporarily alter the activity of many organ systems. Furthermore, heavy alcohol consumption by a pregnant woman can harm her fetus and lead to fetal abnormalities ranging from mild learning deficits to full-blown fetal alcohol syndrome (FAS). Investigating the mechanisms underlying these adverse effects of alcohol consumption in humans frequently is impractical, because alcohol-related disease generally develops only after many years of heavy drinking. Other studies would be unethical to conduct in humans. Therefore, researchers have been forced to use various animal models to gain insight into the processes responsible for alcohol’s effects on the body and to determine new ways of preventing or treating alcohol-related diseases.

This article reviews numerous animal models used to explore alcohol’s effects on several organ systems. First, it provides a brief overview of the animal species and modes of alcohol administration used in such studies. The, article then summarizes the results of studies aimed at identifying the mechanisms underlying alcohol-induced liver damage, alcoholic cardiomyopathy, and FAS.

## Approaches to Studying Alcohol’s Effects on Organ Systems

### Animal Species

To model alcohol’s effects on the human body, researchers ideally wish to use animals closely related to humans, such as nonhuman primates. However, practical and economic considerations generally preclude the use of these animals. In one instance in which primates were used as a model system, however, researchers studied baboons that consumed alcohol with their diets for several years. One-third of these animals eventually developed cirrhosis of the liver (Rubin and Lieber 1974), conclusively demonstrating alcohol’s liver toxicity independent of nutritional factors. To date, however, this experiment represents the only instance in which experimental alcohol administration induced cirrhosis in an animal model. Furthermore, the experiment lasted several years and led to disease development in only a minority of the animals studied. Consequently, the utility of this model for studying the pathogenesis of alcohol-related diseases is limited.

Researchers have used numerous other animals to test alcohol’s effects, most commonly mammals, such as rats, mice, rabbits, hamsters, ferrets, and dogs. Some behavioral and genetic studies have also been conducted in the fruit fly *Drosophila melanogaster* and in the roundworm *Caenorhabditis elegans*. Although the choice of the species depends on the nature of the experiment, most studies have been conducted in rats, owing to their manageable size, ease of handling, low cost, and the availability of extensive scientific data.

#### In Vitro Approaches

Many studies have been conducted in vitro—that is, not with intact animals but with isolated organs, tissues, or cells. For example, researchers have used isolated, perfused organs; tissue slices; suspensions of individual cells; cultures of cells newly isolated from an organism (i.e., primary cell cultures); cells that have gained the ability to grow indefinitely (i.e., transformed cell lines); and isolated cell structures (i.e., organelles).

To study alcohol’s effects on an organ (e.g., the liver or heart), researchers sometimes isolate the particular organ and keep it functional by perfusing it—that is, by passing a fluid or medium through it. Under these conditions, all the different cell types within that organ continue to function in their normal relationships with each other. This is particularly important in organs that comprise several cell types, such as the liver, which contains hepatocytes, endothelial cells, Kupffer cells, stellate cells, and bile duct epithelium, each of which has a specialized role in keeping the liver functioning. By perfusing such organs with fluids containing various alcohol concentrations, researchers can study the coordinated response of all cell types to alcohol. At the same time, the influence of additional factors that might affect organ function in the intact organism (e.g., hormones) is eliminated in the perfused organ. A potential limitation of this approach is that such perfusion experiments last only a few hours before the organ fails.

In cell culture systems, cells derived from mammalian organs (e.g., the liver or brain) or transformed cell lines (e.g., cells derived from certain tumors) are exposed to alcohol to study the cells’ responses to this agent. In such systems, investigators can easily manipulate the cells’ external environment (e.g., the culture medium or the temperature), thereby facilitating analyses of the interactions between alcohol and other molecules, such as hormones, neurotransmitters, and cytokines. In addition, the cultured cells are free from other influences, such as blood flow or the effects of neighboring cells of the same cell type or of another cell type in the same organ. This high degree of control over the environment allows researchers to measure the intracellular responses of individual cells to perturbing agents, such as alcohol. Such analyses frequently involve staining the cells with suitable dyes and then viewing the cells under powerful microscopes.

### Modes of Alcohol Administration in Animal Models

The most physiological methods used to study alcohol’s effects involve acute or chronic alcohol administration to intact animals. Acute alcohol administration is a straightforward procedure during which alcohol is injected into the abdomen (i.e., intraperitoneally) or delivered via a feeding tube directly into the stomach (i.e., intragastrically). In some studies, researchers have also injected alcohol into the veins.

Chronic alcohol exposure of animals poses a greater challenge (for a review of the chronology of the development of various animal models in alcohol research, see Simpson and Peters 1993). In general, chronically exposed animals receive alcohol either with their diets (i.e., orally), intragastrically, or by inhalation.

#### The Liquid Diet Model

The simplest method for providing alcohol chronically is through the drinking water. However, factors such as the animals’ aversion to alcohol, dehydration resulting from insufficient fluid intake, nutritional imbalance, and low blood alcohol concentrations (BACs) resulting from low consumption make this method unsuitable for many types of studies. To address these issues, DeCarli and Lieber (1967) developed a liquid diet that was offered to rats as the only source of food and water (also see Lieber et al. 1989). In this diet, alcohol constitutes 36 percent of the total calories, with protein, carbohydrate, and fat accounting for 18, 11, and 35 percent of total calories, respectively. Although rats generally dislike alcohol, when given a choice of consuming the alcohol-containing diet or starving, they choose the former and consume 14–18 grams of alcohol per kilogram bodyweight (g/kg) per day, usually over a period of 4–6 weeks.

This treatment regimen induces the earliest form of liver damage (i.e., fatty liver, which is characterized by fat deposits throughout the liver) and damage to a key type of liver cells (i.e., the hepatocytes), which can be detected with an electron microscope. The treatment does not, however, lead to the more serious forms of liver damage observed in humans, such as death of liver tissue (i.e., necrosis), inflammation, or scarring of the liver (i.e., fibrosis) (DeCarli and Lieber 1967). However, baboons fed a modified Lieber-DeCarli liquid diet for several years did develop cirrhosis (Rubin and Lieber 1974). Since its development, the Lieber-DeCarli model has been widely used for studies of alcohol’s effects on a variety of other organs, including the heart, brain, and pancreas.

#### The Intragastric Model

Tsukamoto and coworkers (1986) developed the intragastric model for inducing liver damage in rats, based on the hypothesis that rats have a higher rate of alcohol breakdown (i.e., metabolism) than do humans and may require sustained higher BACs than do humans to induce liver damage. In this model, alcohol in a liquid diet (i.e., the Tskukamoto-French diet) is infused directly into the stomach for several months using a surgically implanted catheter. This procedure achieves BACs of 50–80 millimolar (mM) (230–370 mg/dL) and thus exceeds the BACs of 20–40 mM (90–180 mg/dL) obtained with the Lieber-DeCarli model. Rats treated with alcohol using this method develop fatty liver, localized necrosis, and mild portal fibrosis (i.e., fibrosis in the vicinity of small bile ducts through which the liver secretes bile into the gallbladder and small intestine) (Tsukamoto et al. 1990), but do not develop cirrhosis or other irreversible changes.

The model’s ability to reflect alcohol’s effect on the human liver, however, is uncertain. For example, one mechanism through which alcohol induces liver damage is by increasing oxidative stress in the cells—that is, by producing increased amounts of highly reactive, oxygen-containing molecules (i.e., oxygen radicals) or by reducing the levels of antioxidants that scavenge those radicals. (For more information on oxidative stress, oxygen radicals, and antioxidants, see the sidebar, p. 96.) The Tsukamoto-French diet has a high content of unsaturated fats and iron as well as a low content of carbohydrates, a combination that generates substantial oxidative stress, thereby exacerbating the alcohol-induced oxidative stress. Although such considerations point to potential problems in extrapolating accurately from experimental data obtained in animal models to human alcoholic liver disease, such models do provide important information regarding the mechanisms underlying alcohol’s damaging effects at the cellular level.

**Oxidation and Formation of Free Radicals**

In order to function, the body, including each individual cell, requires large amounts of energy. This energy is supplied through the breakdown (i.e., metabolism) of nutrients, such as carbohydrates (e.g., sugars and starch), proteins, and fats. Numerous chemical pathways exist through which nutrients and other molecules (including alcohol) can be broken down. Many of these pathways include chemical reactions that involve oxygen or hydrogen atoms. These reactions are called oxidation reactions. Generally speaking, oxidation reactions are those that add oxygen to or remove hydrogen from a substance (or both). Thus, the metabolism of alcohol also involves two oxidation reactions. First, the enzyme alcohol dehydrogenase converts alcohol to acetaldehyde by removing hydrogen. Then, the enzyme aldehyde dehydrogenase converts acetaldehyde to acetate by removing additional hydrogen and adding oxygen.

Oxidation reactions, however, sometimes result not only in the formation of stable, nontoxic molecules but also generate highly unstable (i.e., reactive) molecules. Many of these molecules contain oxygen and are called oxygen radicals. Common oxygen radicals include super-oxide (O_2_^•^), hydrogen peroxide (H_2_O_2_), and hydroxyl radicals (^•^OH). If unchecked, these oxygen radicals can damage cells by attacking vital cell components, such as fat and protein constituents of the cell wall and the cell’s genetic material.

Because the formation of oxygen radicals is a natural process that occurs during many metabolic processes, cells have developed several protective mechanisms to eliminate those radicals before they can do damage and to prevent their formation. These mechanisms employ molecules called antioxidants, which can inhibit oxidation. Some antioxidants are enzymes found in the cells that steer the radicals into chemical reactions that generate nontoxic molecules. For example, the enzyme superoxide dismutase helps convert superoxide radicals into water. Other antioxidants are compounds found in foods or generated by the body itself, such as vitamin E, vitamin C, and glutathione (GSH). These compounds have several mechanisms of action. For example, GSH can neutralize oxygen radicals by transferring hydrogen to the reactive molecules.

Using their internal antioxidants, cells can deal with normal levels of oxygen radical formation. When oxygen radical formation is greater than normal, or antioxidant levels are lower than normal, however, oxidative stress occurs that may contribute to cell death (i.e., necrosis) and tissue damage, for example, in the liver. Chronic alcohol consumption can increase oxidative stress through several mechanisms. For example, animal models have demonstrated that alcohol metabolism is associated with the generation of oxygen radicals and that chronic alcohol consumption reduces the levels of antioxidant enzymes as well as of other antioxidants (e.g., GSH) (Colell et al. 1998; Nanji and Hiller-Sturmhöfel 1997).

—Biddanda C. Ponnappa and Emanuel Rubin

ReferencesColellAGarcia-RuizCMirandaMSelective glutathione depletion of mitochondria by ethanol sensitizes hepatocytes to tumor necrosis factorGastroenterology115154115511998983428310.1016/s0016-5085(98)70034-4NanjiAAHiller-SturmhöfelSApoptosis and necrosis: Two types of cell death in alcoholic liver diseaseAlcohol Health & Research World214325330199715706744PMC6827678

More recently, Enomoto and colleagues (1999) reported a new rat model in which female Wistar rats received 5 g/kg alcohol intragastrically every 24 hours. After 4 weeks, this treatment induced fat accumulation (i.e., steatosis), inflammation, and necrosis in the liver. Other researchers, however, still must reproduce and confirm this model to establish its validity.

#### The Inhalation Model

The administration of alcohol vapor has been used mostly to study behavioral changes associated with alcohol withdrawal (Goldstein and Pal 1971); however, it has not been widely accepted for inducing organ damage. One drawback of the inhalation model is that it generates a nutritional imbalance between alcohol-exposed and control animals, because intoxicated animals consume less food than do control animals.

## Animal Models of Alcohol-Induced Liver Damage

The liver, which plays a vital role in maintaining the internal environment (i.e., the homeostatic balance) in higher organisms, is one of the primary organs affected by alcohol abuse. Alcohol affects the liver both acutely and after long-term alcohol exposure. In humans, depending on the extent of alcohol abuse, alcoholic liver disease progresses through various stages, from fatty liver to alcoholic hepatitis and finally to cirrhosis. However, the fact that only 15–20 percent of heavy drinkers develop cirrhosis suggests that other risk factors in addition to alcohol play a role in the progression of the disease.

Studies in experimental animals other than primates have failed to produce either alcoholic hepatitis or cirrhosis, limiting their usefulness in studying disease progression. Nevertheless, animal studies have provided data on several interrelated mechanisms that may contribute to the development of alcoholic liver disease (Crabb 1993).

### Alcohol Metabolism

**Figure 1:**
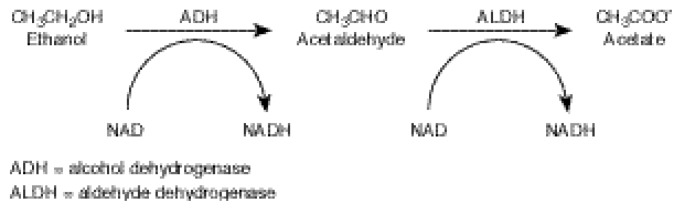
The metabolism of alcohol by the alcohol dehydrogenase pathway. In the liver, alcohol is converted to acetaldehyde, and the acetaldehyde is converted into acetate. The enzyme alcohol dehydrogenase (ADH) assists the chemical reaction in (i.e., catalyses) the first half of alcohol metabolism, and the enzyme aldehyde dehydrogenase (ALDH) catalyzes the second half. Nicotinamide adenosine dinucleotide (NAD) is a co-enzyme that plays an accessory role in the reactions and accepts hydrogen atoms.

The liver is the primary site of alcohol metabolism in the body. The main pathway of alcohol metabolism involves chemical reactions that are mediated by two enzymes (see figure 1 below). First, the enzyme alcohol dehydrogenase (ADH) converts alcohol to acetaldehyde, a highly reactive and toxic compound. Then, the enzyme aldehyde dehydrogenase (ALDH) converts acetaldehyde to acetate, which can be used as a fuel by the cell. During each of these steps, hydrogen atoms are transferred from the alcohol and acetaldehyde (i.e., hydrogen “donor” molecules) to a hydrogen “acceptor” molecule called nicotinamide adenine dinucleotide (NAD), which then becomes reduced NAD (i.e., NADH). This chemical reaction is called an oxidation of alcohol and acetaldehyde. The extent of alcohol metabolism through this pathway is controlled by the ADH content of the liver and by the NADH/NAD ratio (also called the redox state) in the cell. The capacity of this pathway of alcohol metabolism is not influenced significantly by the level or duration of alcohol exposure.

A secondary pathway of alcohol metabolism involves an enzyme system called the microsomal ethanol-oxidizing system (MEOS), which helps eliminate many toxic compounds from the body and uses an enzyme called cytochrome P450 (CYP_2E1_). This enzyme, which converts alcohol to acetaldehyde and uses nicotinamide adenine dinucleotide phosphate (NADP) as a hydrogen acceptor, is induced by chronic alcohol exposure (Lieber and DeCarli 1970). Moreover, CYP_2E1_ is presumed to facilitate alcohol elimination, particularly at the high BACs that prevail in heavy drinkers. Studies in rats found that in the presence of “free iron” (i.e., iron that is not bound to other molecules), CYP_2E1_ also generates various reactive oxygen radicals that can damage cellular components (Cederbaum 1987). Chronic alcohol consumption can substantially increase iron levels in the body. In fact, almost one-third of alcoholics have excessive iron levels in their livers, much of which is free iron (Nanji and Hiller-Sturmhöfel 1997). Thus, these elevated iron levels may contribute to alcoholic liver damage.

**Figure 2:**
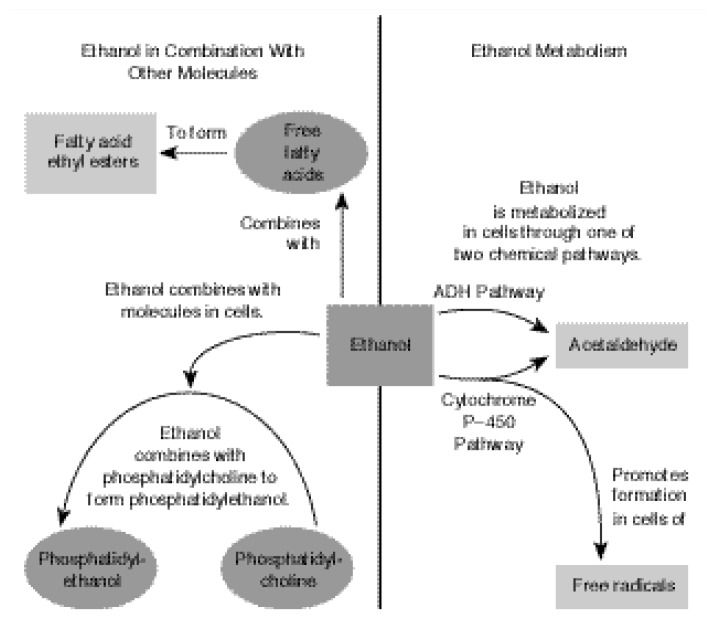
The presence of alcohol (i.e., ethanol) in tissues results in the generation of potentially toxic metabolites in the cells. Ethanol molecules combine with free fatty acids to form fatty acid ethyl esters, which have been linked to tissue injury. Ethanol also may combine with phosphatidylcholine (i.e., a membrane component) to form phosphatidylethanol, a compound that potentially alters cell membranes. Ethanol metabolism forms acetaldehyde and, potentially, free radicals, which may interact with cellular components and possibly interfere with their functions.

Most acetaldehyde generated by ADH and CYP_2E1_ is rapidly converted to acetate by ALDH. Some acetaldehyde, however, may combine with liver proteins to form harmful compounds that can impair the function of various cellular components and enzymes. In addition, alcohol can combine with other molecules in the cell to form potentially dangerous compounds, such as fatty acid ethyl esters and phosphatidylethanol (see figure 2). As described in the section “Animal Models of Alcoholic Cardiomyopathy,” pp. 98–100, these compounds may damage the membrane that surrounds each cell, thereby contributing to alcoholic heart disease.

### Generation of Oxygen-Derived Free Radicals

Oxidative stress may also play a role in the pathogenesis of alcoholic liver disease, and several mechanisms contribute to the generation of excessive oxygen radicals or the reduction of antioxidant levels (Ishi et al. 1997). The primary organelles that generate oxygen radicals during alcohol metabolism are microsomes and mitochondria. For example, rat liver microsomes treated with alcohol generate a free radical, identified as 1-hydroxyethyl radical, that can damage proteins as well as DNA. The alcohol-inducible enzyme CYP_2E1_ appears to play an important role in the increased production of this radical, because radical formation was much higher in microsomes isolated from alcohol-fed rats (which showed elevated CYP_2E1_ activity) than in microsomes isolated from control rats (see Ishi et al. 1997 and references therein).

Studies on alcohol metabolism using perfused rat livers have noted an increased production of a certain oxygen radical called superoxide anion (O_2_^•^) (Bautista and Spitzer 1992). This radical is generated mainly in the process that produces ATP, the cell’s universal energy currency, in the mitochondria. Acute alcohol administration can increase superoxide generation in liver mitochondria (Sinaceur et al. 1985), and alcohol-fed animals develop structurally and biochemically abnormal mitochondria. These mitochondria have decreased levels of proteins needed for ATP production and oxidize various compounds at reduced rates.

The mitochondria also contain the enzyme superoxide dismutase (SOD), which generates hydrogen peroxide radicals. Normally, this radical is degraded by a mitochondrial enzyme called glutathione peroxidase. In the presence of iron, however, some of the hydrogen peroxide generates other highly reactive radicals that in turn can impair both the structure and function of the mitochondria. As mentioned above, iron levels frequently are elevated in alcoholics. Furthermore, acute alcohol administration induces SOD activity and may, therefore, promote tissue damage (see Ishi et al. 1997).

Another mechanism through which alcohol-induced oxidative stress might result in liver disease involves the enhanced metabolism of fat molecules (i.e., lipid peroxidation) (Muller and Siles 1987). Free radicals generated from oxidative stress can remove electrons from unsaturated fatty acids, resulting in lipid radicals. The lipid radicals can react with oxygen to form lipid peroxide radicals, which, in turn, interact with other fatty acids, thereby creating a chain reaction of lipid peroxidation. Such chain reactions may generate biologically active compounds, such as molecules that cause widening or narrowing of blood vessels, thereby increasing the risk of cardiovascular disease. Moreover, lipid peroxidation may generate molecules that recruit inflammatory cells (i.e., chemoattractant molecules) rendering the host more susceptible for inflammatory attacks.

As discussed in the sidebar (see p. 96), the body has developed several protective systems (i.e., antioxidants) that eliminate radicals, including glutathione (GSH) and vitamin E, as well as certain enzymes. When these protective systems are overwhelmed, tissue damage from free radicals may result. Animal studies found that both acute and chronic alcohol exposure can interfere with antioxidant activity. For example, large acute doses of alcohol reduce GSH levels in the liver by 25–50 percent (Crabb 1993), and GSH levels in the mitochondria of chronically alcohol-fed animals are also reduced. Similarly, long-term alcohol administration has been shown to decrease vitamin E levels in rat liver mitochondria. Thus, a reduction in antioxidant levels caused by chronic alcohol intake predisposes the liver (and possibly other organs) to attack by free radicals.

### Endotoxins

Another mechanism that may contribute to alcohol-related liver damage involves bacterial endotoxins, which are molecules derived from the cell walls of certain bacteria, including many that live in the intestine. When those bacteria die, endotoxins are released and may penetrate the intestinal wall to enter the bloodstream, potentially causing various symptoms of infection. Through the bloodstream, the endotoxins eventually are transported to the liver, where they are ingested (i.e., phagocytosed) predominantly by specialized cells (i.e., the Kupffer cells) and eliminated from the body. The interaction between endotoxins and Kupffer cells (and possibly other phagocytic cells in the spleen) triggers the release of certain molecules (i.e., inflammatory cytokines) that serve to control the damage caused by endotoxin (e.g., tumor necrosis factor alpha [TNF–α] and prostaglandins). An overproduction of TNF–α may damage hepatocytes.

Both human alcoholics and experimental animals exposed to alcohol exhibit increased levels of endotoxin and inflammatory cytokines circulating in the blood (Nanji et al. 1993). This alcohol-induced increase in endotoxin levels may result from an enhanced “leakiness” (i.e., permeability) of the intestinal wall and possibly from a reduced capacity to eliminate bacterial endotoxin. Animal studies using intra-gastric alcohol administration have also demonstrated that Kupffer cells, particularly with their production of prostaglandin E (PGE_2_) and TNF–α, contribute to endotoxin-mediated liver damage associated with alcohol intake (Thurman et al. 1998). One hypothesis suggests that the excess Kupffer cell-derived PGE_2_ stimulates oxygen consumption in the hepatocytes in certain areas of the liver of alcohol-fed animals. This increased oxygen consumption may promote a temporary lack of oxygen (i.e., hypoxia) in the blood flowing through those liver areas, followed by a return to normal oxygen levels in the liver. Such hypoxic episodes, in turn, might promote the generation of free radicals.

### Liver Regeneration

The liver’s ability to regenerate itself is essential for allowing the organ to recover from various forms of injury, including those related to alcohol. For example, liver regeneration may help explain why only a relatively small proportion of drinkers develop irreversible liver disease. By the same token, inhibition of liver regeneration by alcohol abuse may be an important factor in the progression of liver disease in humans. To study this issue, researchers surgically removed parts of the livers of alcohol-exposed and control rats. These studies found that both acute and chronic alcohol administration inhibit the production of new genetic material (i.e., DNA) in the regenerating liver (Yoshida et al. 1997). Without sufficient DNA production, however, the remaining liver cells cannot multiply as rapidly to regenerate the liver. Other researchers (Zeldin et al. 1996) have investigated the effects of acute and chronic alcohol administration on cytokine-inducible transcription factors. These transcription factors are regulatory proteins that are activated by cytokines and which are required for the conversion of genetic information into functional proteins during various processes, including liver regeneration. The investigators observed that chronic alcohol administration impeded the ability of the cytokines to activate the transcription factors, thereby limiting the generation of new liver cells.

### Signal Transduction in the Liver

Alcohol intake can affect an organ indirectly by stimulating the release of hormones and other chemical messengers that influence a variety of cellular processes or by promoting exposure to harmful substances such as endotoxins. In addition, alcohol can directly affect cells by interacting with and damaging components of the cell membrane (proteins and fat molecules [i.e., lipids]). This membrane also contains numerous docking molecules (i.e., receptors) that serve as point of contact for chemical messengers involved in cellular communication, such as hormones. This interaction initiates mechanisms that translate chemical messages arriving at the cell membrane into cascades of biochemical reactions within the cell, eventually resulting in changes in gene activity in the cell’s DNA. This process is known as signal transduction. Using animal model systems, researchers have shown that alcohol profoundly affects these communication processes (Hoek et al. 1992). For example, acute alcohol exposure can activate several signal transduction systems, thereby initiating a plethora of reactions within the cell. Conversely, prolonged alcohol exposure can cause the cells to become desensitized to the chemical messengers and impair cell activity.

## Animal Models of Alcoholic Cardiomyopathy

**Figure 3:**
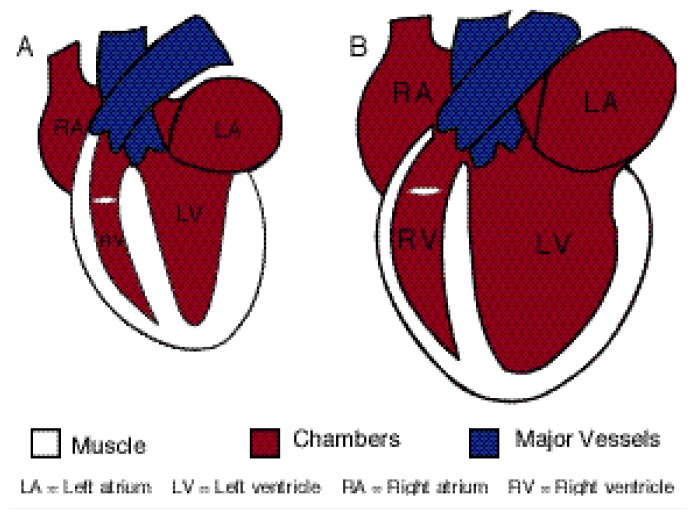
Schematic illustration of (A) a normal heart and (B) a heart with alcoholic cardiomyopathy. Both hearts are shown in their state at the end of contraction (i.e., at end-systole).

Another organ affected by alcohol abuse is the heart. The typical form of alcoholic heart disease, called dilated cardiomyopathy, is a degenerative disease of the heart muscle (i.e., myocardium). It is characterized by a reduced capacity of the heart to pump blood (i.e., depressed cardiac output), reduced ability of the heart muscle to contract, widening (i.e., dilatation) of all heart chambers, and various other changes that occur on a cellular level (see figure 3). There are practical obstacles to studying the pathogenesis of this disorder in human patients, especially with obtaining tissue samples for such analyses. Some of these problems can be resolved using animal model systems (Richardson et al. 1998; Thomas et al. 1994), (For information on a chicken model of alcoholic cardiomyopathy, see the article in this issue by Tabakoff and Hoffman, pp. 77–84). Researchers have not been able, however, to reproduce true dilated cardiomyopathy in animals. Furthermore, both in humans and in experimental animals, studies on alcohol’s direct effects on the heart in the intact organism are complicated by alcohol’s indirect effects on other organ systems and molecules that modulate heart function. For example, alcohol induces changes in the levels of catecholamines—an important class of chemical compounds, including dopamine, norepinephrine, and epinephrine, that are involved in normal cellular communication and which influence cardiac function.

Alcohol’s direct effects on the heart can best be studied by studying perfused hearts or isolated cardiac muscle preparations. In studies using perfused hearts, alcohol depressed a variety of indicators of the heart’s ability to contract. For example, the rate and extent to which pressure developed in the heart chambers decreased and the rate at which the heart muscle subsequently relaxed slowed down (Lochner et al. 1969; Segel 1988). Furthermore, in hearts perfused with alcohol, researchers observed an increased volume of blood in the heart’s left ventricle at the end of the heartbeat, when the chambers are relaxed (i.e., the diastole) (Segel 1988). These data suggest that alcohol interferes directly with the contractile function of the heart muscle.

### Mechanisms of Alcohol’s Actions on the Myocardium

Animal studies have identified several mechanisms through which alcohol might affect the myocardium. For example, studies in rats demonstrated that acute alcohol intoxication can rapidly lead to a reduction in muscle protein production (i.e., synthesis). Specifically, acute alcohol exposure reduced the synthesis of myofibrillar protein, which is required for heart muscle contraction (Richardson et al. 1998). Moreover, chronic alcohol exposure led to a loss of myofibrillar protein after 6 weeks. These findings are consistent with studies showing that myofibrillary protein loss occurred in human alcoholics and that the remaining cardiac myofibrillary protein was in disarray (Hibbs et al. 1965).

Acute alcohol treatment also might reduce the heart’s ability to contract by interfering with the process through which nerve signals induce the muscle to contract. For most muscles to contract, the brain emits a signal that is transmitted to nerve cells (i.e., neurons) that are in contact with the muscle cell. When receiving the signal, these neurons release a neurotransmitter (usually acetylcholine) that interacts with receptors on the muscle-cell surface. This interaction causes a tiny electrical impulse at the muscle cell membrane that through a process called excitation-contraction (E–C) coupling, activates proteins within the cell that perform the actual contraction (i.e., contractile proteins). Calcium ions in the fluid filling the cell (i.e., the cytosol) play a key role in E–C coupling. When the cell “rests,” calcium levels in the cytosol are low. The electrical impulse at the cell membrane, however, causes the release of large amounts of calcium from storage compartments within the cell. This sudden increase in calcium levels allows the contractile proteins to perform their function, leading to muscle contraction. To release the contraction and allow the muscle to relax again, the calcium is rapidly transported back into the storage compartments, resulting in a return of the calcium concentration to its normal, low levels. This temporary increase in calcium levels followed by a rapid decrease is called a calcium transient.

Alcohol may interfere with this E–C coupling by affecting the calcium transients (Thomas et al. 1994). Methods for measuring calcium in the cytosol of intact cells have allowed researchers to examine alcohol’s effects on the calcium transients in isolated cardiac muscle cells (i.e., myocytes) and muscle fibers. Such studies have demonstrated that moderate to high levels of alcohol reduce the magnitude of the change in calcium levels in the cytosol in response to an electrical impulse (Thomas et al. 1994). In addition, other studies have shown that muscle contraction is impaired by alcohol concentrations that do not affect calcium levels in the cytosol (Guarnieri and Lakatta 1990). These findings suggest that in addition to affecting calcium transients, alcohol reduces the sensitivity of the contractile proteins to calcium.

### Effects of Long-Term Alcohol Administration on Cardiac Function

The effects of long-term alcohol consumption on cardiac function have been examined in a wide variety of animal models, including mice, rats, dogs, monkeys, and baboons. Most of those studies found that although metabolic changes occur within a few weeks of alcohol administration, signs of depressed contractile function are detectable only after several months of alcohol consumption. Various studies found the following effects of long-term alcohol consumption:

- Rats receiving drinking water containing 30 percent alcohol water for 8 months exhibited an increase in the size of the left ventricle, indicating an enlargement of the heart. At the cellular level, prolonged alcohol administration led to a reduction in the total number of myocytes comprising the left ventricle (see Thomas et al. 1994 and references therein).
- The left ventricle of dogs that received alcohol for 18 months displayed increased scarring of the heart tissue as evidenced by increases in total collagen levels, which may reduce the heart’s ability to extend and contract during each heart beat (Thomas et al. 1980).
- Detailed studies of the structure of cardiac myocytes in alcohol-fed animals found various structural changes that are similar to some defects found in human patients with alcoholic cardiomyopathy.

### Effects of Alcohol Metabolism on the Heart

Animal model systems have also helped elucidate the metabolic effects of long-term alcohol exposure on cardiac function (Thomas et al. 1994). For example, compared with normal heart muscle, alcoholic heart muscle may have a reduced capacity for oxidative metabolism—that is, the process that generates energy by breaking down the sugar glucose. Alternatively, alcoholic heart muscle may generate additional energy in the absence of oxygen (i.e., nonoxidatively) by breaking down glycogen (i.e., a storage form of glucose in the cell) to lactic acid. Such a change in metabolism may explain the more acidic intracellular environment observed when the workload is increased in perfused hearts of alcohol-fed hamsters compared with control animals. Furthermore, alcohol metabolites also may adversely affect cardiac function, as follows:

- Acetaldehyde may affect cardiac function indirectly by enhancing the release of catecholamines.
- Fatty acid ethyl esters, which accumulate in the hearts of alcohol-fed animals, may damage mitochondria and alter the properties of biological membranes (e.g., membranes surrounding the cells or various organelles) (Laposata 1998), thereby impairing cell function.
- Phosphatidylethanol can also disrupt membrane functions (Hoek et al. 1992).

### Contractile Function

Long-term alcohol consumption also interferes with the heart’s contractile function (Thomas et al. 1994). For example, in dogs, long-term alcohol feeding lowered the rate with which pressure developed in the left ventricle and increased pressure in the left ventricle at the end of the diastole. Researchers made similar observations in rats. Moreover, dogs that were fed alcohol for 1 year developed inefficient, random contractions (i.e., fibrillation) in the ventricle more easily than did control animals that received no alcohol. As in humans, acute alcohol administration to animals that had previously consumed alcohol chronically further increased the animals’ vulnerability to fibrillation.

Heart function has also been examined in isolated heart muscles derived from alcohol-fed animals. In isolated muscles from the left ventricle of rats that had been fed alcohol for 5 weeks to several months, one or more indicators of the heart’s mechanical performance were significantly reduced. In studies using isolated perfused hearts, however, a species difference existed with regard to the effects of long-term alcohol feeding. Thus, whereas perfused hearts from hamsters exhibited a reduction in pressure during contraction of the heart (i.e., systolic pressure) and an increase in pressure during the relaxation of the heart (i.e., end-diastolic pressure), hearts from rats showed little change in contractile properties after chronic alcohol administration.

In summary, scientists can produce alterations in heart function in animal models that are similar to those found in human alcoholics. Animal models do have some limitations, however. For example, researchers have not been able to reproduce alcoholic cardiomyopathy with its reduced output. Moreover, in contrast with alcoholic cardiomyopathy in humans, all cardiac effects observed in animal models can be reversed by withdrawing the alcohol.

## Animal Studies of FAS

In humans, alcohol exposure during gestation has been associated with a variety of negative outcomes, including cognitive and behavioral deficits, compromised growth, and death during late pregnancy or soon after birth (i.e., in the perinatal period) (Streissguth et al. 1980). The most severe manifestation of prenatal alcohol exposure is FAS, which is characterized by a recognizable cluster of facial malformations (i.e., facial dysmorphology), growth retardation, and mental retardation. Animal studies on FAS have advanced researchers’ knowledge in these areas, as well as of the effects of prenatal alcohol exposure on the immune system, hormonal (i.e., endocrine) systems, and central nervous system. Most of these studies have been conducted in mice and rats that received alcohol, although researchers have used other species as well (Guerri 1998; Hannigan 1996).

Some problems exist when using animal models to investigate alcohol’s effects on the developing fetus. For example, the gestational process differs somewhat among different species. Thus, the major growth spurt in the human fetal brain occurs during the third pregnancy trimester, whereas the corresponding growth spurt in mice and rats occurs after birth. Researchers must consider such differences when designing experiments and alcohol administration schedules. Nevertheless, as described in the following sections, alcohol’s effects on the fetus are qualitatively similar in humans and experimental animals.

### Prenatal and Postnatal Growth Retardation

Babies born to alcohol-abusing women frequently have a lower than normal birth weight. Theoretically, this growth retardation could result from malnutrition of the mother rather than from prenatal alcohol exposure itself, because alcohol-abusing women are less likely to eat a nutritious diet during pregnancy. Researchers have investigated this issue in rats and mice, using various modes of alcohol administration. The resulting findings suggest that fetal alcohol exposure per se rather than maternal malnutrition causes reduced birth weight (see Norton and Kotkoshie 1991 and references therein).

### Facial Dysmorphology

The distinctive facial features associated with FAS include small eye openings, a small nose, a flattened groove between the upper lip and the nose (i.e., philtrum), and an abnormally small upper jaw (i.e., maxillary hypoplasia). To determine whether these features could be reproduced in an animal model, researchers administered alcohol by a stomach tube to pregnant monkeys once a week during gestation. The offspring of these animals exhibited several of these FAS characteristics (i.e., a small nose, poorly defined philtrum, and maxillary hypoplasia) (Clarren and Bowden 1982). Similar observations were made in mouse embryos exposed to alcohol at a specific time during gestation (Sulik et al. 1981). These findings confirm the observations in humans that alcohol exposure during pregnancy can produce the characteristic facial dysmorphology in the developing fetus.

Researchers also have begun to identify the mechanisms underlying alcohol’s effects on facial characteristics, such as alcohol-induced cell death. Studies in mouse and chick embryos revealed that this cell death primarily occurs in specific groups of cells, particularly in a group called neural crest cells (Smith 1997). A subset of these cells forms various facial structures, such as the jaws, nose, ears, orbital bone around the eye, and forehead. When chick embryos were exposed to alcohol during a specific (and very short) time period early during embryo development, cell death occurred among the neural crest cells, resulting in cell loss and, consequently, facial malformation (Smith 1997). In the neural crest cells, alcohol may exert its deleterious effects by interfering with the formation of a substance called retinoic acid, a vitamin A derivative that is necessary for the development of those cells into facial features (National Institute on Alcohol Abuse and Alcoholism [NIAAA] 2000).

### Immune System Deficiencies

Another consequence of prenatal alcohol exposure is impaired immune function in children with FAS, as evidenced by their increased risk for infections. Accordingly, researchers have examined alcohol’s effects on the developing immune system using animal models. These studies found that alcohol exposure in mice during the perinatal period was associated with reduced function of the thymus, a gland located near the base of the neck in which certain immune cells called T-lymphocytes mature and become functional (Ewald and Frost 1987). T-lymphocytes play a central role in regulating the body’s immune response to various pathogens. This function depends on the cells’ ability to divide rapidly during the immune response in order to produce more T-lymphocytes. Consequently, impaired function of the thymus and the resulting reduction in functional T-lymphocytes can render the organism susceptible to infections. Moreover, prenatal alcohol exposure may impair T-lymphocyte function because, in rats exposed to alcohol during gestation, T-lymphocytes in the spleen no longer responded to substances that induce cell division (i.e., mitogens), thereby limiting the body’s ability to respond adequately to infections (Monjan and Mendel 1980).

### Central Nervous System Abnormalities

Alcohol’s detrimental effect on the developing brain is the most serious feature of FAS. Numerous brain areas are affected by prenatal alcohol exposure. The sensitivity of any area depends on the developmental period during which the alcohol exposure occurs and on the amount of the alcohol dose.

#### Cerebral Cortex

The cerebral cortex is the outer layer of the brain, which is essential for information processing, control of motor function, speech, emotions, behavior, memory, and other critical mental functions. Numerous studies in rats found that alcohol can interfere with the development of the cerebral cortex, as follows:

- Maternal alcohol consumption during the formation of the cerebral cortex results in thinning and disorganization of its outer layers (Norton and Kotkoskie 1991).
- Alcohol exposure from gestational day 6 to the end of gestation alters the final location of neurons that connect the cortical area responsible for the senses and motor activity (i.e., the sensorimotor cortex) with the spinal cord (Miller 1987).
- Prenatal alcohol exposure causes neurons in the frontal cortex to develop fewer extensions that connect each neuron with numerous neighboring neurons (i.e., less dendritic branching). Moreover, the number of dendrites is markedly reduced (Stolenburg-Didinger and Spohr 1983), indicating that communication among neurons, which is critical for “thinking” processes, is impaired.
- Neurons of the parietal cortex show changes in dendrites similar to those found in mentally retarded children.
- Alcohol exposure in the early post-natal period results in a decreased number of synapses in the cortex, accompanied by increased numbers of vesicles in the remaining synapses. These changes may indicate that the cortical synapses have remained immature, which may result in learning disabilities.

#### Hippocampus

The hippocampus is a brain area associated with learning and memory. One of the major functions of the hippocampus is to construct “spacial cognitive maps” of the surroundings. Mathews and Morrow (2000) recently reviewed the effects of acute and chronic alcohol exposure on the hippocampal function. In rats, gestational alcohol exposure reduced the number of certain neurons in the hippocampus by 20 percent. This reduction may contribute to some of the cognitive deficits observed in alcohol-exposed infants (Barnes and Walker 1981).

#### Cerebellum

The cerebellum is an area located at the base of the brain that is involved in controlling posture, balance, and coordination. Whereas in humans the cerebellum develops largely during the prenatal period, neuron production and development in rats continue into the postnatal period. Consistent with this developmental pattern, alcohol administration to rats during gestation did not alter the number of specialized cells in the cerebellum (i.e., Purkinje cells), although some structural changes occurred in those cells, and the maturation of certain cell components was delayed compared with control rats. These differences, however, disappeared by day 17 after birth. Conversely, alcohol exposure throughout gestation and into the early postnatal period reduced the number of synaptic contacts in a particular subregion of cerebellum (i.e., the molecular layer) and the number of Purkinje cells (Norton and Kotkoskie 1991). These changes may contribute to the impaired motor control (e.g., balance deficits) observed in alcohol-exposed children.

### Neurochemical Alterations

Animal studies have shown that gestational alcohol exposure not only affects neuronal development but also neuronal function, as indicated by alterations in neurotransmitter levels. One important neurotransmitter is acetylcholine, which allows communication among neurons as well as between neurons and muscle cells. Acetylcholine’s actions are regulated by an enzyme called acetylcholinesterase. When rat fetuses are exposed to alcohol during the final week of gestation, post-natal levels of acetylcholinesterase are reduced throughout the brain (Rudeen and Guerri 1985). These results suggest the possibility that prenatal alcohol exposure delays the development of or permanently alters acetylcholine-using neuronal systems in the brain.

Animal studies also found that pre-natal alcohol exposure reduces the levels of the other neurotransmitters. For example, in rats, very early prenatal alcohol exposure significantly delays the development of serotonin-using neurons in many brain regions during gestational periods that are most likely critical for normal brain development (NIAAA 2000). Similarly, prenatal exposure to even moderate alcohol levels causes decreases in the number and function of certain receptors for the neurotransmitter glutamate (NIAAA 2000). If these effects occur during critical periods of brain development, they may contribute to the mental and behavioral deficiencies associated with FAS. Moreover, both prenatal and postnatal alcohol exposure alters the levels of the neurotransmitter gamma-amino butyric acid (GABA), although the direction of these changes varies among different brain regions. Thus, GABA levels increased in some regions (e.g., the frontal cortex and the amygdala), decreased in some regions (e.g., the hippocampus), and remained unchanged in other regions (e.g., the hypothalamus and septal area) (Ledig et al. 1988). In general, alcohol-induced reductions in neurotransmitter levels are most pronounced when alcohol exposure occurs throughout gestation and into the postnatal period (Norton and Kotkoskie 1991).

In addition to alcohol’s direct toxic effects on brain development, indirect effects, such as alcohol-related nutritional deficiencies (e.g., zinc deficiency) may contribute to alcohol’s adverse effects on fetal development (Norton and Kotkoskie 1991). Furthermore, researchers have postulated that alcohol’s toxic effects on the placenta and alcohol-induced fetal hypoxia play a role in the pathogenesis of central nervous system malformations associated with FAS.

In summary, animal experiments have clearly demonstrated that the fetus is susceptible to various types of damage during gestational alcohol exposure and that the extent of damage is related to the dose and extent of alcohol exposure.

## Conclusions

Numerous animal model systems have contributed substantially to an understanding of the pathways by which alcohol might cause organ damage, such as alcoholic liver disease and cardiomyopathy, as well as FAS. Nevertheless, the ability of these models to mirror these conditions in humans is somewhat limited. For example, whereas many alcohol-induced conditions in humans are irreversible (e.g., cirrhosis and cardiomyopathy), researchers have been unable to produce such irreversible organ damage in animal models. One reason for this inability may be that such alcohol-related disorders in humans take many years of alcohol consumption to develop in humans. In addition, differences among species may exist in disease pathogenesis.

Another limitation of animal models is the difficulty in duplicating the circumstances under which alcohol consumption occurs in human alcoholics. Most experimental systems are designed to assess the effects of either acute or chronic alcohol exposure. Acute models analyze the immediate effects of alcohol or its metabolites on cellular function or molecular organization. Such experimental situations may indeed reproduce the effects of acute alcohol intake on human organs and tissues. Conversely, chronic models are concerned with the long-term adaptive or injurious responses of organs and cells to persistent alcohol exposure. Long-term alcohol consumption in humans can vary substantially over time with respect to the amount and frequency of alcohol intake. These variations are difficult to mimic in animals. Furthermore, many of the changes produced by chronic alcohol exposure (e.g., cirrhosis and cardiomyopathy) are reversible in experimental animals, whereas in humans these conditions are permanent. Accordingly, researchers cannot be absolutely certain of the veracity of their models. In addition, it will be difficult to determine whether chronic diseases associated with alcohol abuse in humans reflect the accumulation of numerous acute injuries or whether they are independent of acute effects and caused by as yet unknown factors. Nevertheless, a better understanding of the alcohol-related maladies clearly depends upon researchers’ ability to learn more about the molecular and cellular events associated with alcohol consumption. Some of these events reflect direct interactions between alcohol and various cell constituents (e.g., proteins, fats, and DNA), whereas other effects are indirect, acting through multiple biochemical cascades. All of these interactions are similar in humans and laboratory animals. Thus, although a need remains for improved animal models that mimic the human conditions more closely, existing models are capable of providing considerable information regarding the mechanisms by which alcohol injures the body.

## Footnotes

1 For a definition of this and other technical terms used throughout this issue, please see the central glossary, pp. 141–143.
